# A qualitative study of facilitators and barriers to implementing worksite policies that support physical activity

**DOI:** 10.1186/s12889-018-6045-x

**Published:** 2018-09-27

**Authors:** Maryanne M Bailey, Rachel K Coller, Keshia M Pollack Porter

**Affiliations:** 1 The Johns Hopkins Hospital, 600 N. Wolfe Street Osler Building #720, Baltimore, MD 21287 USA; 2 Johns Hopkins Bloomberg School of Public Health, Baltimore, MD 21205 USA; 3 Department of Health Policy and Management, Johns Hopkins Bloomberg School of Public Health, 624 N. Broadway, #380A, Baltimore, MD 21205 USA

**Keywords:** Physical activity, Workplace wellness, Policy

## Abstract

**Background:**

Physical inactivity is associated with several chronic diseases that are costly to society, employers, and individuals. Workplaces are a common location for physical activity (PA) initiatives because of the amount of time individuals who are employed full time spend at work. This research examined a statewide worksite wellness program, the Healthiest Maryland Businesses (HMB) program, to fill an important gap regarding the facilitators for and barriers to implementing workplace policies that support PA.

**Methods:**

Individual telephone interviews were conducted in December 2015 with six HMB Coordinators and their supervisor, and from August through October 2016 with a purposeful sample of 15 businesses of various sizes from across Maryland, to learn about the role of leadership, and successes and challenges of implementing PA programs and policies. The sample of businesses was intentionally selected to capture perspectives from a range of businesses. Interviews were recorded and professionally transcribed. Descriptive coding was used to identify dominant themes that addressed the study aims and research questions.

**Results:**

PA was not described as a priority for several large and small businesses. To garner more support for PA, interviewees emphasized associating PA initiatives with measures the businesses care about, such as health care costs from claims data. Small businesses also described having a need for PA programming yet reported having significant resource constraints. There was a strong interest in developing guidance for implementing PA break policies, which was mentioned as a critical support for workplace PA promotion. More commitment and investment of resources from leadership, and an engaged wellness committee with company representation at all levels and roles, were identified as vital for impactful programs.

**Conclusions:**

Most businesses are implementing PA programs with limited policy supports, which was mentioned as a barrier. Successful implementation of workplace wellness programs broadly, and PA initiatives specifically, are achievable through leadership buy-in, employee input, and policy supports, along with highlighting the economic benefits for businesses.

## Background

Physical inactivity is associated with increased rates of coronary heart disease, type 2 diabetes, breast cancer, colon cancer, stroke, and reduced life expectancy [1, 2]. Despite this, only about half of American adults meet the federal guidelines for physical activity (PA) [3]. By 2023, chronic diseases among the aging workforce is projected to cost about $4.2 trillion annually [4]. Estimates are that an employee with cardiovascular disease costs the employer nearly 60 h and over $1100 more in lost productivity a year than an employee without cardiovascular disease [5]. Increasing PA among adults has the potential to lessen the impact of these non-communicable diseases on individuals and employers [1, 2].

Since many adults who are employed full time spend most of their waking hours at work, some scholars describe workplaces as an optimal location to implement health promotion and wellness initiatives [6]. Workplace wellness interventions that address risk factors for chronic conditions such as PA, nutrition, and obesity have been shown to positively influence worker absenteeism, stress, and job satisfaction [7]. The Guide to Community Preventive Services reported strong evidence of a consistent effect of worksite PA and nutrition programs on employee weight and body mass index [8]. In 2014, 73% of small employers (3–199 employees) and 98% of large employers (200 or more) offered at least one wellness program [9]. There is evidence that these programs can also result in cost savings for companies [6–8], which further supports their benefits for businesses [6].

Several studies have documented specific components of workplace wellness programs, which vary widely in size and components. For example, the Centers for Disease Control and Prevention identified common elements of workplace health programs to include health education classes and access to local fitness facilities [9]. Also, Pronk & Kottke identified the following criteria that have proven relevant in the implementation of employer-sponsored programs and influenced employers to invest in health initiatives: use existing employee relationships between the individual and the environment; employ evidence-based interventions; and create programs that are relevant to the needs of the workplace and fit within a comprehensive structure [10].

Studies have also documented barriers and facilitators of employee participation in worksite wellness programs [11]. Workplace programs that specifically address PA are most effective, in terms of participation, when they have clear goals related to the business’s objectives, clear and effective communication, employee involvement in planning processes, incentives, and support from higher management [12]. Facilitators, such as policies that define times in which employees can be physically active during the workday are also correlated with PA [13]. A mixed-methods telephone-based study conducted in four metropolitan areas found that access to 11 worksite supports, such as incentives for walking and/or bicycling to work and access to an indoor exercise facility, increased the odds of meeting PA recommendations through leisure-time PA [14]. While these data provide guidance on the types of workplace strategies to implement, there are clear gaps in the literature regarding implementation, beyond questions about reach and participation.

The Maryland Department of Health created Healthiest Maryland Businesses (HMB) - a worksite wellness program supported by a DP13–1305 grant from the Centers for Disease Control and Prevention. The HMB program is a statewide program that creates a culture of wellness at work – an environment that makes the healthiest choice the easiest choice. The objectives of this study were to examine how program coordinators of a state health department led workplace wellness program support businesses in promoting PA in the workplace, and examine the facilitators for and barriers to implementing impactful PA programs and policies for a sample of businesses involved in the HMB program. This research is significant as it fills an important gap in understanding the facilitators and barriers that workplaces face when implementing PA programs and policies.

## Methods

### Program description

The HMB program, with over 400 businesses as of 2017, had several goals: to raise awareness about the importance of a healthy workforce, recruit business leaders who will support healthy policies in the workplace, recognize this important commitment and their successes, and improve Maryland businesses’ bottom line [15]. Regional HMB coordinators utilized the Centers for Disease Control and Prevention’s (CDC) Worksite Health ScoreCard (HSC), which is an assessment tool for employers to use to prevent and reduce heart disease, stroke, & related health conditions among their employees [16, 17]. After the HSC baseline assessment of each business, the HMB coordinator would set wellness goals for each business based on the HSC findings and work with a regional HMB technical assistance coordinator to execute their goals. All employers with Maryland-based businesses are eligible to become HMB members at no cost, but are required to complete the HSC. Data from the HMB program indicate that in Maryland specifically, employees spend 9.2 h at work on average per day, which makes the workplace an ideal setting for health promoting activities [18].

This research utilized qualitative data collection methods to examine implementation of workplace PA initiatives among HMB businesses. Separate semi-structured interview guides were developed by the research team to collect information from those who oversee and implement the HMB program (i.e., HMB Coordinators and their supervisor) and those who are HMB program members (i.e., businesses in the HMB program). A systematic document review of relevant materials collected from the interviewees was also conducted.

### HMB coordinators

During December 2015, one-on-one telephone interviews, lasting around 30 min in length, were conducted by two of the authors, who are experienced interviewers, with all six HMB Coordinators to understand: their role and the types of assistance and resources provided to businesses in the program, how they use the CDC HSC, and what they perceive as facilitators and barriers for PA initiatives. The HMB Coordinators’ supervisor was interviewed after the six coordinators, which allowed us to confirm and clarify information provided from the other interviews.

### HMB case studies

At the time this research began, there were about 200 businesses in the HMB program. We decided a priori to interview 15 businesses based on the time and resources available to our study team. We aimed to purposively select the 15 business to obtain a diverse sample that was representative of the overall population of HMB businesses.

Businesses selected for case study interviews were identified in two ways. They either self-selected by indicating on questions added to the survey when the CDC HSC was completed that they could be contacted by researchers for further study, or they were identified by their HMB coordinator for further study due to success with some aspect of PA initiatives in the workplace.

Specifically, we reviewed the 26 businesses in the program who completed the CDC HSC and responded affirmatively to a question about willingness to be considered for this research. Of those 26, six did not provide contact information to reach them. The other 20 businesses were stratified by size, type, and county. Thirteen additional business were suggested by HMB coordinators during their interviews and considered for inclusion in the sample.

Of the initial 20, 11 were interviewed, 7 did not respond, and 2 did not follow up to schedule an appointment. Zero businesses actively declined to be interviewed. Of the 13 additional businesses suggested by HMB coordinators, 6 were selected by the study team to be contacted because they could fill gaps in representation from businesses of certain size, type, and geographic location; 4 of the 6 were interviewed. The other 2 businesses were not contacted because the HMB coordinator who referred them did not provide us the contact information or respond to requests for contact information. The Results section contains a table that summarizes the included worksites, except for the county where the business was located to ensure that the businesses were not identifiable.

From August through October 2016, one-on-one telephone interviews about 30 min in length were conducted with one individual from each of 15 businesses who was responsible for employee health and wellness initiatives. Interviewees were probed about their role, the role of leadership, existing PA programs and policies, associated successes and challenges, resources, and evaluation. In some instances, the respondent was a human resources (HR) employee, and in other instances the respondent was a non-HR employee who was specifically hired to manage employee health and wellness.

Before the interviews concluded, we asked each respondent if they would share any documents relevant to their PA program and policies. We only received documents from a couple of worksites, which two members of the study team read for any pertinent information related to the interview questions. Review of these documents did not yield additional useful information.

### Coding and data analysis

All interviews were audio recorded and transcribed verbatim by a professional transcription company. A deductive approach, informed by the study’s research questions, aims, and objectives, was used to initially develop the codes. This approach to developing the codes for two coding dictionaries, one for the HMB Coordinator interviews and another for the HMB businesses, ensured that the resulting codes would be responsive to the study aims.

One investigator read all 7 transcripts, from the interviews of the 6 HMB Coordinator and their supervisor and developed a list of preliminary codes based on the research questions, emerging themes, and subthemes. The study’s PI also read each of the transcripts and added a few new codes that emerged from this process. Both investigators independently coded two transcripts and together compared them to determine the reliability of the codes by assessing differences in applying the codes and to better standardize code application by clarifying code definitions. The codes were revised, and the two investigators recoded the initial subset of transcribed interviews to create the final codebook. One investigator coded the remaining 5 transcripts using that codebook.

For the 15 case study transcripts, one investigator read through 3 randomly selected interview transcripts and developed a list of preliminary codes, again based on research questions, emerging themes, and subthemes. All three investigators independently coded one transcript and together compared them, which resulted in one addition and one change to the codebook. Three additional transcripts were independently coded by two investigators and together compared to determine the reliability of the codes using the same process described above to create the final codebook. The transcripts were entered into the QSR NVivo data management program. One investigator independently coded the remaining 11 interviews with subsequent review by a second investigator.

The study PI led the analysis of the codes, which involved descriptive coding to “attribute a class of phenomena to a segment of text” [19]. This process led to understanding how the interviewees viewed the HMB coordinator’s roles, assistance and resources provided to HMB businesses, perspectives regarding the CDC HSC, and facilitators and barriers for PA initiatives in the workplace. The results are presented by these key themes. After analysis was complete, member checking was used to validate the data by sending a summary of the findings to the interviewees.

The Institutional Review Boards at the Johns Hopkins Bloomberg School of Public Health and the Maryland Department of Health approved this research.

## Results

### HMB coordinators

HMB coordinators each supported an average of 50 businesses across Maryland. All coordinators completed the Certified Worksite Wellness Specialist Program through the National Wellness Institute, and some coordinators mentioned completing the CDC Work@Health training. Coordinators described how the CDC HSC data helped them to assess priority areas for businesses and connect them with resources based on their specific needs. Among the key themes that emerged from the coordinators and their supervisor were that small businesses have the greatest need and resource constraints, and are therefore more challenging to assist.

PA was not a priority for many businesses they supported. To address this barrier, there was an expressed need to better communicate about how PA impacts measures the businesses care about, such as absenteeism rates, and costs from claims data with the understanding that it may take a few years to see return-on-investment (ROI). To promote PA to the businesses, the coordinators relied on existing resources available on the Internet from organizations such as the CDC and the American Heart Association.

Another challenge cited was that grant funds for the HMB program had aims that sometimes differed from businesses’ needs and priorities. For example, HMB grant funds aimed to increase PA and walking, but some businesses prioritized improving nutrition and tobacco cessation. Merging those different priorities and providing information for PA/walking as a priority was a challenge the coordinators tried to address.

A few coordinators noted that some businesses wanted to be held up as an example of leadership for wellness. Being a leader in employee wellness is something the businesses could use for their own marketing and perhaps distinguish them from their competitors. Coordinators also noted that local health departments could serve as a model for what other businesses can and should do regarding policies for PA breaks during the work day. For example, if health departments are encouraging businesses to allow PA breaks during work time, but the health department itself does not have that policy for their own employees, businesses may be less likely to implement it.

### HMB case studies

Table 1 describes the 15 businesses that participated in a case study interview. In addition to size and type, the businesses were also categorized by county/region because we aimed to have a representative sample from rural/suburban/urban areas. Of the 15 businesses, 6 were small businesses (of which 1 was for profit, 2 were non-profit/government, 3 were non-profit/other); 4 were medium-sized businesses (of which 1 was for profit, 2 were non-profit/government, 1 was non-profit/other); and 5 were large businesses (of which 2 were for profit, 0 were non-profit/government, 3 were non-profit/other). As reflected in Table 1, most businesses were small- and large-sized, non-profit/other businesses from the health care and social service sector serving rural areas.

**Table 1:** Description of businesses included in the qualitative analysis (*n* = 15)

Number of Employees	Sector	Type of Business	Geographic Area
Small (< 200)			
6	Health Care and Social Service	Non-Profit/Other	Rural
10	Construction	For Profit	Suburban
45	Health Care and Social Service	Non-Profit/Other	Rural
69	Health Care and Social Service	Non-Profit/Government	Rural
94	Health Care and Social Service	Non-Profit/Government	Rural
173	Professional, Scientific and Technical Services	Non-Profit/Other	Suburban
Medium (200–550)			
242	Transportation and Warehousing	For Profit	Rural
288	Health Care and Social Service	Non-Profit/Government	Rural
331	Health Care and Social Service	Non-Profit/Government	Urban
535	Utilities	Non-Profit/Other	Rural
Large (551+)			
1000	Accommodation and Food Services	For Profit	Suburban
1267	Health Care and Social Assistance	Non-Profit/Other	Rural
3000	Manufacturing	For Profit	Urban
6917	Health Care and Social Assistance	Non-Profit/Other	Suburban
8000	Educational Services	Non-Profit/Other	Urban

Key findings from the interviews are summarized in Table 2. Respondents consistently identified role modeling on the part of leadership as a facilitator for PA policies and programs. Having commitment and investment from leadership was identified as essential to the success of any PA policies and programs. For example, one interviewee shared a perspective that we heard from others we spoke with:

“The programs that are most successful are the ones that senior representation, senior management really embraces and talks up. That helps raise the awareness. We’ll send out emails, talk in meetings about upcoming wellness initiatives, be it a program, a contest, but when senior management reinforces it and follows up with their own email or they talk about it, that always helps generate more interest and excitement in doing it. I mean, really their influence is very important.”

**Table 2:** Key themes from the interviews with case study participants (*n* = 15)

Commonly Cited Worksite PA^a^ Activities	Walking meetings, walking groups/clubs, gym membership discounts, one-time classes, wellness challenges, use of PA trackers
Barriers to PA Initiatives	- Limited resources, particularly for small businesses - Many workplaces find it difficult to make PA a priority - Burdensome to implement PA break policies - HMB grant aims do not always coincide with the priorities and needs of participating businesses - Lack of support from HMB business leadership - Extreme weather poses a challenge for outdoor PA - Physical limitations for gym equipment, space to conduct workout classes, access to stairs and shower facilities - Workplace culture is not viewed as a place to promote PA (e.g., viewed PA as a personal issue rather than a company one) - Low rates of participation in PA programming among workers - Logistics of certain businesses prevent programming and activities—liability associated with PA, government organizations cannot offer certain participation incentives
Facilitators of PA Initiatives	- Leadership support for PA programs is vital to success - Offering programs at a convenient time for employees helps to improve participation - Having a budget specifically for wellness activities - Need a wellness committee comprised of employees separate from HR responsible for planning and giving feedback on PA activities - Have tools in the office that could prompt PA among workers such as hula hoops and jump ropes - Offer a wide range of activities to attract a wider range of employees - Encourage employees to share PA success stories - Putting policies in writing improves uptake and participation - Some employees are more motivated to participate in PA programs when incentives are offered - Employees are motivated by different things, therefore communication advertising PA activities should address these motivators

^a^*PA* physical activity

This point was emphasized by another interviewee who stated:

“Our CEO participates when we have different challenges, and he runs the 5Ks and 10Ks with the employees. He definitely is a big part of the getting that support from the top down. It is very important, and we do have that so I think it is a key part of why we’re so successful. It’s been reiterated that the supervisors need to provide support because (the program takes place) during work time. So we have had to have management reminded that they are to be supportive of the wellness program.”

Interviewees also shared their perspectives on what has worked and what some challenges have been. An engaged wellness committee with company representation at all levels and roles was also identified as vital, particularly for enacting PA break policies and impactful programs. A few participants mentioned that incentives and competitions are useful especially when trying to get employees to try new things and to engage new people. However, employees may stay engaged longer if they also contribute something to participate, for example, a small fee to participate in a fitness class. Respondents from larger companies that were able to subsidize employee costs to participate in fitness programs and or gym memberships did not think the issue was cost, but supporting the right programs. One respondent captured this well and said: “I think it’s a matter of finding the right programs to increase the participation.”

Businesses like to know what other similar type businesses are doing that works, and a newsletter was suggested as one way of disseminating that information. Highlighting businesses, through recognition and awards, for being leaders in employee health can motivate similar type businesses to be competitive and initiate their own PA initiatives.

Key themes identified as challenges involved certain types of businesses that have unique challenges to implementing PA policies and programs because of unusual work hours, shifts, working off-site, and limitations to what can be worn in the work environment (i.e., no Fitbits, smartphones, or pedometers). For a small subset of businesses that operated in around the clock shifts, finding the right time to hold fitness classes was a challenge. There were challenges engaging part-time and hourly employees who work in the field. Some businesses, such as those in hospitality, don’t have all employees under one roof and experience high turnover as well.

Small businesses generally have unique challenges related to a lack of funding, access to resources, and a limited number of employees. Small businesses also described challenges with having the physical space to support activity. For example, the small businesses in our sample did not have stairs to promote stair use. Other businesses with stairs noted the lack of showers for people to use after doing things like stair walking, which when done at a good pace could make someone sweat.

Most PA policies were informal and indicated that PA initiatives take place on employees’ time including during their lunchtime. This becomes a convenience issue for many employees. Additionally, staff employees who are responsible for PA initiatives in the workplace have limited time, multiple responsibilities, and few resources. Even for businesses that have a formal PA policy in place that allows time during work hours to engage in PA, there are challenges. For example, these policies typically require that an employee’s work not fall behind, and he/she first obtain permission from the supervisor, some of whom may be more supportive of the policy than others. Some employees may be perceived as not having enough work to do if they are able to take a 30-min PA break during the workday. One non-profit government business reported that only about 20 out of 285 employees utilized the policy because of these barriers. The policy should be consistently implemented across departments, divisions, and supervisors with enforcement from management/leadership in order to address some of these barriers. One other policy challenge, noted by small workplaces, is how a policy that encourages walk breaks can become a burden for a small business if there are only a few employees doing the work. These small businesses are looking for guidance as to how to reduce this burden and still promote PA.

The importance of evaluation was also discussed by the interviewees. Tracking the number of employees who partake in PA initiatives and satisfaction surveys were the most frequent types of evaluation used in this subset of businesses. Some workplaces surveyed employees about what types of activities they would participate in. There was expressed interest in learning more about how to evaluate policies and programs and any tools to guide this process, especially because of lack of capacity or understanding regarding the best ways to evaluate workplace health promotion initiatives. Businesses requested access to template surveys and an outline of how to evaluate their efforts (formative, process, and program evaluation); these were perceived to be valuable tools. There were also requests for trainings through webinars showing businesses how to conduct evaluations of policies and programs and to identify evidence-based PA programs.

## Discussion

This qualitative study explored, with a subset of businesses enrolled in a worksite wellness program, the barriers and facilitators to implementing PA policies and programs. Additionally, the coordinators who are charged with assisting these businesses with their initiatives provided their insights into the barriers and facilitators to PA in the workplace. Some insights gleaned support previous findings from other studies. Other findings provide more specific detail and examples from the in-depth, semi-structured interviews.

Very few workplaces in our sample had a formal written policy that encouraged PA breaks during work hours. The absence of such a policy was described by respondents as a barrier to employees being more active because a perception was that employees may not feel empowered to take time from their work day to be active. These findings support the results of a meta-analysis of workplace PA initiatives completed in 2009, which found that interventions that allowed employees to participate during paid-time had a greater impact on employee health [7]. Specifically, interventions with employees during paid time reported larger mean effect sizes than those with employees receiving interventions outside company paid time on both fitness and anthropometric measures. Additionally, interventions with employee interventionists were more effective than those with others as interventionists for improved fitness, lipid profile, and anthropometric measures [7]. Since only a few businesses had policies, we were unable to explore barriers to implementing those specific policies; our discussions ended up focusing more on implementing workplace wellness programs. Future research is needed to further understand how workplace policies that support PA are being implemented.

Business size was the main driver of differences in workplace programs and policies. Small businesses face challenges related to lack of funding, physical space, shower facilities, and access to stairs and gym membership discounts. Interviewees perceived that something as simple as creating a policy to allow time for PA breaks could become a burden for small businesses. The Community Guide recommends point-of-decision prompts to encourage individuals to use stairs [20], which is not always feasible for small businesses that may be located on a single level. Small businesses should consider supporting other strategies to incorporate PA into the day, such as walking meetings, when feasible [21].

Businesses reported wanting more guidance and tools to conduct evaluation beyond just employee satisfaction surveys, especially small businesses and non-profits. Employers desire evidence-based supports for increasing PA [14]. The type of incentives used is important to increasing PA. Some incentives were counter to wellness, for example, winning a parking space closer to the building as a prize in a PA competition. Incentives that are provided for activities that increase PA, such as an incentive for using public transportation, should be utilized. Multiple worksite supports for PA have been shown to increase the odds that employees meet PA recommendations [14].

These data also support sharing evaluation data with leadership and management at all levels, as well as the employees. Businesses should evaluate their PA initiatives and use the data to guide future policy and program development and implementation [22]. Demonstrating a potential return on investment has also been recognized in prior studies as a key factor to support workplace initiatives that support PA [14]. Individuals included in this research emphasized that having data on ROI could convince business leaders of the impact PA policies and programs can have on their bottom line. For businesses that lack expertise or the capacity to do their own evaluation, they could connect with academic institutions where researchers and students could help them evaluate their data.

The main limitations of this study relate to selection and response biases. The businesses were not randomly selected for this research. We initially selected businesses from the ones that agreed to be considered for this research. Moreover, eleven businesses contacted to participate either did not respond or schedule an interview within the study timeframe. Businesses that chose not to participate could be different from businesses that participated in terms of challenges to implementing PA policies and programs in the workplace. As a result, although we sought a representative sample, the 15 businesses included in this research may not be representative of all businesses enrolled in the HMB program, thereby limiting the transferability of these results. Additionally, we spoke with one key informant from each business relying on the perspective of a single respondent, who may have not been fully aware of all PA-related activities. We believe we interviewed the person most knowledgeable about each worksite’s PA initiatives, but we cannot confirm this was the case.

## Conclusions

Worksite wellness programs can help businesses create effective policies and programs through leadership buy-in, appropriate incentives, employee input, and development of tools to evaluate and modify policies and programs to meet employee and business priorities. Companies could also benefit from having policies that support engaging in PA during work hours. These policies should be put in writing and consistently implemented across the business by all supervisors. Challenges that are specific to business size and type require creative strategies to facilitate PA, and when possible, specific resources should be created to support small businesses, especially in rural areas that may lack access to community assets that support PA.

## Abbreviations

CDC: Centers for Disease Control and Prevention
HMB: Healthiest Maryland Businesses
HR: Human Resources
HSC: Worksite Health ScoreCard
PA: Physical Activity
ROI: Return-on-Investment
